# *trans*-Bis[bis­(2-methoxy­phen­yl)phenyl­phosphine-κ*P*]dichloridopalladium(II)

**DOI:** 10.1107/S1600536809040744

**Published:** 2009-10-10

**Authors:** Charmaine van Blerk, Cedric W. Holzapfel

**Affiliations:** a University of Johannesburg, Department of Chemistry, PO Box 524, Auckland Park, Johannesburg 2006, South Africa

## Abstract

The structure of the title compound, [PdCl_2_(C_20_H_19_O_2_P)_2_], shows a square-planar geometry for the Pd^II^ ion within a Cl_2_Pd[PPh(PhOMe)_2_]_2_ ligand set. The Pd^II^ atom sits on an inversion centre and therefore the asymmetric unit contains the Pd^II^ atom, one Cl atom and one bis­(2-methoxy­phen­yl)phenyl­phosphine ligand. The *trans* arrangement of ligands is also imposed by symmetry.

## Related literature

For related structures of similar palladium complexes and their use in methoxy­carbonyl­ation reactions, see: Robertson & Cole-Hamilton (2002 ▶); Van Leeuwen *et al.* (2003 ▶); Williams *et al.* (2008 ▶).
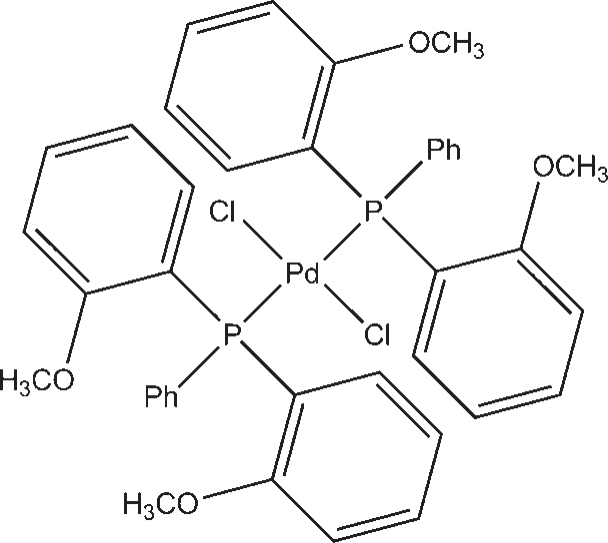


## Experimental

### 

#### Crystal data

[PdCl_2_(C_20_H_19_O_2_P)_2_]
*M* *_r_* = 821.94
Monoclinic, 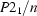

*a* = 9.1617 (2) Å
*b* = 12.7203 (3) Å
*c* = 16.4939 (4) Å
β = 94.114 (1)°
*V* = 1917.24 (8) Å^3^
*Z* = 2
Mo *K*α radiation
μ = 0.75 mm^−1^
*T* = 296 K
0.22 × 0.18 × 0.12 mm

#### Data collection

Bruker SMART CCD diffractometer
Absorption correction: multi-scan (**APEX* AXScale*; Bruker, 2008 ▶) *T*_min_ = 0.853, *T*_max_ = 0.916
23435 measured reflections
4750 independent reflections
3339 reflections with *I* > 2σ(*I*)
*R*_int_ = 0.064

#### Refinement

*R*[*F* ^2^ > 2σ(*F* ^2^)] = 0.036
*wR*(*F* ^2^) = 0.089
*S* = 1.02
4750 reflections
225 parameters
H-atom parameters constrained
Δρ_max_ = 0.63 e Å^−3^
Δρ_min_ = −0.38 e Å^−3^

### 

Data collection: *SMART-NT* (Bruker, 1999 ▶); cell refinement: *SAINT* (Bruker, 2008 ▶); data reduction: *SAINT*; program(s) used to solve structure: *SHELXS97* (Sheldrick, 2008 ▶); program(s) used to refine structure: *SHELXL97* (Sheldrick, 2008 ▶); molecular graphics: *X-SEED* (Barbour, 2001 ▶) and *Mercury* (Macrae *et al.*, 2006 ▶); software used to prepare material for publication: *publCIF* (Westrip, 2009 ▶).

## Supplementary Material

Crystal structure: contains datablocks I, global. DOI: 10.1107/S1600536809040744/bh2252sup1.cif

Structure factors: contains datablocks I. DOI: 10.1107/S1600536809040744/bh2252Isup2.hkl

Additional supplementary materials:  crystallographic information; 3D view; checkCIF report

## Figures and Tables

**Table:** 

P1—Pd1	2.3458 (6)
Cl1—Pd1	2.3048 (7)

**Table:** 

Cl1—Pd1—P1	93.24 (2)
Cl1^i^—Pd1—P1	86.76 (2)

Symmetry code: (i) 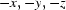.

**Fig. 1.**
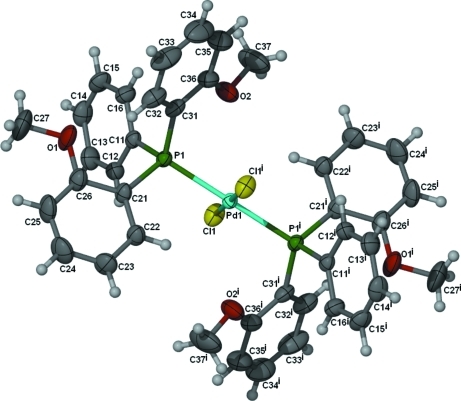
Molecular structure of (I) with displacement ellipsoids drawn at the 50% probability level. Atoms labelled with (i) are at symmetry position (-x, -y, -z).

## supplementary crystallographic information

### Comment

The palladium-catalysed methoxycarbonylation (Robertson & Cole-Hamilton, 2002) of 1-alkenes is an active area of research. The preferred (pre)-catalysts of general structure (Ar_3_P)_2_Pd*X*_2_ (*X* = Cl, DMS, OTf, *etc*.) are either preformed or generated *in situ*. The X-ray structures (Van Leeuwen *et al.*, 2003; Williams *et al.*, 2008) of several of this class of palladium(II) complexes have been determined. Only some of these have found application in the catalysis of the methoxycarbonylation reaction, but their use results mainly in the formation of linear esters (Robertson & Cole-Hamilton, 2002). However, we have identified some palladium(II) complexes which catalyse the regioselective formation of branched esters. We report here the structure of one of these.

The structure of the title compound, [PdCl_2_(C_40_H_38_P_2_O_4_)], shows a square planar geometry for the Pd^II^ ion within the Cl_2_(PPh(PhOMe)_2_) ligand set. The palladium atom sits on a centre of inversion and therefore the asymmetric unit contains the palladium atom, one chlorine atom and one bis-(2-methoxyphenyl)phenylphosphine ligand. Figure 1 shows the molecular structure of the title compound.

### Experimental

Bis-(2-methoxyphenyl)phenylphosphine (1.288 g, 4 mmol) was added to a solution of palladium(II) chloride (354 mg, 2 mmol) and anhydrous lithium chloride (168 mg, 4 mmol) in methanol (15 ml). The mixture was refluxed in an atmosphere of nitrogen until all the phosphine reagent had reacted and a light yellow product had formed (*ca.* 45 min). The reaction mixture was cooled and the product collected by filtration, washed with fresh methanol and dried under vacuum. The crude product (1.37 g) was dissolved in dichloromethane and crystallization of the title compound was carried out by diethyl ether vapour diffusion into the dichloromethane. The crystals of the title compound were pale yellow blocks (m.p. > 503 K, decomp.) and a suitable crystal was selected for the single-crystal X-ray diffraction analysis.

### Refinement

H atoms were geometrically positioned and refined in the riding-model approximation, with C—H = 0.93–0.97 Å, and *U*_iso_(H) = 1.2*U*_eq_(C) or 1.5*U*_eq_(C). The highest peak in the final difference map is 2.20 Å from H37A and the deepest hole is 0.31 Å from Pd1.

### Crystal data

**Table:**

[PdCl_2_(C_20_H_19_O_2_P)_2_]	*F*(000) = 840
*M**_r_* = 821.94	*D*_x_ = 1.424 Mg m^−^^3^
Monoclinic, *P*2_1_/*n*	Mo *K*α radiation, λ = 0.71073 Å
Hall symbol: -P 2yn	Cell parameters from 5078 reflections
*a* = 9.1617 (2) Å	θ = 2.5–24.7°
*b* = 12.7203 (3) Å	µ = 0.75 mm^−^^1^
*c* = 16.4939 (4) Å	*T* = 296 K
β = 94.114 (1)°	Block, yellow
*V* = 1917.24 (8) Å^3^	0.22 × 0.18 × 0.12 mm
*Z* = 2	

### Data collection

**Table:**

Bruker SMART CCD diffractometer	4750 independent reflections
Radiation source: fine-focus sealed tube	3339 reflections with *I* > 2σ(*I*)
graphite	*R*_int_ = 0.064
φ and ω scans	θ_max_ = 28.3°, θ_min_ = 2.0°
Absorption correction: multi-scan (*APEX AXScale*; Bruker, 2008)	*h* = −12→12
*T*_min_ = 0.853, *T*_max_ = 0.916	*k* = −16→16
23435 measured reflections	*l* = −21→21

### Refinement

**Table:**

Refinement on *F*^2^	Primary atom site location: structure-invariant direct methods
Least-squares matrix: full	Secondary atom site location: difference Fourier map
*R*[*F*^2^ > 2σ(*F*^2^)] = 0.036	Hydrogen site location: inferred from neighbouring sites
*wR*(*F*^2^) = 0.089	H-atom parameters constrained
*S* = 1.02	*w* = 1/[σ^2^(*F*_o_^2^) + (0.0441*P*)^2^] where *P* = (*F*_o_^2^ + 2*F*_c_^2^)/3
4750 reflections	(Δ/σ)_max_ = 0.001
225 parameters	Δρ_max_ = 0.63 e Å^−^^3^
0 restraints	Δρ_min_ = −0.37 e Å^−^^3^
0 constraints	

### Fractional atomic coordinates and isotropic or equivalent isotropic displacement parameters (Å^2^)

**Table:**

	*x*	*y*	*z*	*U*_iso_*/*U*_eq_	
C11	0.0228 (3)	0.1927 (2)	−0.15944 (16)	0.0323 (6)	
C12	0.0094 (3)	0.1268 (2)	−0.22659 (16)	0.0401 (7)	
H12	−0.0432	0.0646	−0.2240	0.048*	
C13	0.0734 (3)	0.1526 (3)	−0.29741 (18)	0.0505 (8)	
H13	0.0647	0.1072	−0.3417	0.061*	
C14	0.1500 (3)	0.2454 (3)	−0.3024 (2)	0.0528 (8)	
H14	0.1927	0.2628	−0.3501	0.063*	
C15	0.1626 (3)	0.3112 (3)	−0.2373 (2)	0.0505 (8)	
H15	0.2128	0.3744	−0.2409	0.061*	
C16	0.1016 (3)	0.2850 (2)	−0.16577 (18)	0.0426 (7)	
H16	0.1136	0.3299	−0.1213	0.051*	
C21	−0.2607 (3)	0.1459 (2)	−0.10562 (17)	0.0363 (6)	
C22	−0.3400 (3)	0.0578 (2)	−0.08536 (18)	0.0434 (7)	
H22	−0.2954	0.0076	−0.0508	0.052*	
C23	−0.4839 (3)	0.0426 (3)	−0.1152 (2)	0.0569 (9)	
H23	−0.5358	−0.0163	−0.1002	0.068*	
C24	−0.5481 (4)	0.1156 (3)	−0.1671 (2)	0.0670 (10)	
H24	−0.6444	0.1055	−0.1876	0.080*	
C25	−0.4735 (4)	0.2040 (3)	−0.1898 (2)	0.0631 (10)	
H25	−0.5191	0.2525	−0.2254	0.076*	
C26	−0.3300 (3)	0.2201 (3)	−0.15904 (19)	0.0472 (8)	
C27	−0.3017 (6)	0.3742 (4)	−0.2385 (3)	0.1117 (19)	
H27A	−0.3180	0.3354	−0.2882	0.167*	
H27B	−0.2311	0.4286	−0.2454	0.167*	
H27C	−0.3921	0.4052	−0.2245	0.167*	
C31	−0.0464 (3)	0.2692 (2)	−0.00191 (17)	0.0403 (7)	
C32	−0.1582 (4)	0.3370 (2)	0.0142 (2)	0.0518 (8)	
H32	−0.2510	0.3276	−0.0114	0.062*	
C33	−0.1318 (5)	0.4201 (3)	0.0691 (2)	0.0707 (11)	
H33	−0.2066	0.4664	0.0796	0.085*	
C34	0.0048 (6)	0.4326 (3)	0.1071 (2)	0.0805 (13)	
H34	0.0212	0.4869	0.1444	0.097*	
C35	0.1175 (5)	0.3675 (3)	0.0916 (2)	0.0737 (12)	
H35	0.2099	0.3779	0.1175	0.088*	
C36	0.0933 (4)	0.2854 (2)	0.03693 (19)	0.0505 (8)	
C37	0.3458 (4)	0.2356 (4)	0.0401 (3)	0.1033 (17)	
H37A	0.3705	0.3048	0.0224	0.155*	
H37B	0.4060	0.1847	0.0154	0.155*	
H37C	0.3618	0.2312	0.0981	0.155*	
O1	−0.2494 (2)	0.30626 (18)	−0.17624 (15)	0.0639 (7)	
O2	0.1969 (2)	0.21487 (18)	0.01702 (15)	0.0628 (7)	
P1	−0.06893 (7)	0.15556 (5)	−0.06890 (4)	0.03007 (16)	
Cl1	0.12498 (8)	−0.06305 (6)	−0.10623 (4)	0.04534 (19)	
Pd1	0.0000	0.0000	0.0000	0.02642 (9)	

### Atomic displacement parameters (Å^2^)

**Table:**

	*U*^11^	*U*^22^	*U*^33^	*U*^12^	*U*^13^	*U*^23^
C11	0.0277 (13)	0.0311 (14)	0.0378 (14)	0.0047 (11)	0.0009 (11)	0.0057 (11)
C12	0.0412 (16)	0.0401 (16)	0.0384 (16)	−0.0050 (13)	−0.0002 (13)	0.0031 (13)
C13	0.056 (2)	0.059 (2)	0.0359 (16)	0.0035 (16)	0.0020 (15)	0.0028 (15)
C14	0.0448 (19)	0.069 (2)	0.0461 (18)	0.0056 (17)	0.0128 (15)	0.0177 (17)
C15	0.0432 (18)	0.0463 (19)	0.063 (2)	−0.0063 (15)	0.0130 (16)	0.0168 (16)
C16	0.0434 (17)	0.0356 (16)	0.0492 (17)	−0.0033 (13)	0.0056 (14)	0.0043 (13)
C21	0.0268 (14)	0.0406 (16)	0.0415 (15)	0.0064 (12)	0.0030 (12)	0.0073 (12)
C22	0.0336 (16)	0.0468 (18)	0.0492 (17)	0.0012 (13)	0.0001 (13)	0.0083 (14)
C23	0.0321 (17)	0.062 (2)	0.076 (2)	−0.0062 (15)	−0.0007 (16)	0.0080 (19)
C24	0.0341 (18)	0.091 (3)	0.074 (2)	0.0017 (19)	−0.0093 (17)	0.004 (2)
C25	0.0407 (19)	0.080 (3)	0.067 (2)	0.0151 (18)	−0.0095 (17)	0.022 (2)
C26	0.0381 (17)	0.0505 (19)	0.0529 (18)	0.0081 (14)	0.0027 (14)	0.0131 (15)
C27	0.128 (4)	0.086 (3)	0.114 (4)	−0.004 (3)	−0.035 (3)	0.063 (3)
C31	0.0531 (18)	0.0263 (14)	0.0425 (16)	0.0021 (12)	0.0100 (15)	0.0037 (12)
C32	0.062 (2)	0.0367 (17)	0.060 (2)	0.0031 (15)	0.0236 (17)	0.0025 (15)
C33	0.099 (3)	0.039 (2)	0.079 (3)	0.009 (2)	0.046 (2)	−0.0043 (19)
C34	0.124 (4)	0.054 (3)	0.064 (3)	−0.007 (3)	0.016 (3)	−0.018 (2)
C35	0.102 (3)	0.049 (2)	0.068 (2)	−0.010 (2)	−0.014 (2)	−0.0157 (19)
C36	0.067 (2)	0.0352 (16)	0.0484 (18)	−0.0089 (15)	−0.0040 (16)	−0.0039 (14)
C37	0.059 (3)	0.094 (3)	0.151 (4)	−0.009 (2)	−0.033 (3)	−0.026 (3)
O1	0.0490 (13)	0.0574 (14)	0.0837 (17)	0.0081 (11)	−0.0064 (12)	0.0361 (13)
O2	0.0521 (14)	0.0522 (14)	0.0802 (16)	0.0009 (11)	−0.0218 (12)	−0.0140 (12)
P1	0.0294 (3)	0.0265 (3)	0.0342 (4)	0.0024 (3)	0.0015 (3)	0.0036 (3)
Cl1	0.0549 (5)	0.0435 (4)	0.0395 (4)	0.0124 (3)	0.0169 (3)	0.0021 (3)
Pd1	0.02524 (14)	0.02514 (14)	0.02887 (14)	0.00229 (12)	0.00193 (10)	0.00150 (12)

### Geometric parameters (Å, °)

**Table:**

C11—C16	1.386 (4)	C27—O1	1.400 (4)
C11—C12	1.387 (4)	C27—H27A	0.9600
C11—P1	1.827 (3)	C27—H27B	0.9600
C12—C13	1.383 (4)	C27—H27C	0.9600
C12—H12	0.9300	C31—C32	1.379 (4)
C13—C14	1.379 (4)	C31—C36	1.405 (4)
C13—H13	0.9300	C31—P1	1.822 (3)
C14—C15	1.361 (5)	C32—C33	1.402 (5)
C14—H14	0.9300	C32—H32	0.9300
C15—C16	1.382 (4)	C33—C34	1.368 (6)
C15—H15	0.9300	C33—H33	0.9300
C16—H16	0.9300	C34—C35	1.362 (6)
C21—C22	1.390 (4)	C34—H34	0.9300
C21—C26	1.410 (4)	C35—C36	1.388 (4)
C21—P1	1.821 (3)	C35—H35	0.9300
C22—C23	1.387 (4)	C36—O2	1.363 (4)
C22—H22	0.9300	C37—O2	1.414 (4)
C23—C24	1.367 (5)	C37—H37A	0.9600
C23—H23	0.9300	C37—H37B	0.9600
C24—C25	1.381 (5)	C37—H37C	0.9600
C24—H24	0.9300	P1—Pd1	2.3458 (6)
C25—C26	1.390 (4)	Cl1—Pd1	2.3048 (7)
C25—H25	0.9300	Pd1—Cl1^i^	2.3048 (7)
C26—O1	1.363 (4)	Pd1—P1^i^	2.3458 (6)
			
C16—C11—C12	117.9 (3)	H27A—C27—H27C	109.5
C16—C11—P1	123.6 (2)	H27B—C27—H27C	109.5
C12—C11—P1	118.5 (2)	C32—C31—C36	119.0 (3)
C13—C12—C11	120.9 (3)	C32—C31—P1	124.0 (3)
C13—C12—H12	119.6	C36—C31—P1	116.9 (2)
C11—C12—H12	119.6	C31—C32—C33	120.0 (3)
C14—C13—C12	120.1 (3)	C31—C32—H32	120.0
C14—C13—H13	119.9	C33—C32—H32	120.0
C12—C13—H13	119.9	C34—C33—C32	119.6 (4)
C15—C14—C13	119.6 (3)	C34—C33—H33	120.2
C15—C14—H14	120.2	C32—C33—H33	120.2
C13—C14—H14	120.2	C35—C34—C33	121.6 (4)
C14—C15—C16	120.6 (3)	C35—C34—H34	119.2
C14—C15—H15	119.7	C33—C34—H34	119.2
C16—C15—H15	119.7	C34—C35—C36	119.5 (4)
C15—C16—C11	120.9 (3)	C34—C35—H35	120.3
C15—C16—H16	119.5	C36—C35—H35	120.3
C11—C16—H16	119.5	O2—C36—C35	124.8 (3)
C22—C21—C26	118.1 (3)	O2—C36—C31	114.9 (3)
C22—C21—P1	118.9 (2)	C35—C36—C31	120.3 (3)
C26—C21—P1	122.9 (2)	O2—C37—H37A	109.5
C23—C22—C21	121.8 (3)	O2—C37—H37B	109.5
C23—C22—H22	119.1	H37A—C37—H37B	109.5
C21—C22—H22	119.1	O2—C37—H37C	109.5
C24—C23—C22	118.9 (3)	H37A—C37—H37C	109.5
C24—C23—H23	120.6	H37B—C37—H37C	109.5
C22—C23—H23	120.6	C26—O1—C27	119.1 (3)
C23—C24—C25	121.6 (3)	C36—O2—C37	119.2 (3)
C23—C24—H24	119.2	C21—P1—C31	108.64 (14)
C25—C24—H24	119.2	C21—P1—C11	103.58 (12)
C24—C25—C26	119.7 (3)	C31—P1—C11	104.58 (13)
C24—C25—H25	120.2	C21—P1—Pd1	108.91 (9)
C26—C25—H25	120.2	C31—P1—Pd1	111.13 (9)
O1—C26—C25	123.7 (3)	C11—P1—Pd1	119.37 (8)
O1—C26—C21	116.2 (3)	Cl1—Pd1—Cl1^i^	180.00 (4)
C25—C26—C21	120.0 (3)	Cl1—Pd1—P1^i^	86.76 (2)
O1—C27—H27A	109.5	Cl1^i^—Pd1—P1^i^	93.24 (2)
O1—C27—H27B	109.5	Cl1—Pd1—P1	93.24 (2)
H27A—C27—H27B	109.5	Cl1^i^—Pd1—P1	86.76 (2)
O1—C27—H27C	109.5	P1^i^—Pd1—P1	180.00 (5)
			
C16—C11—C12—C13	0.2 (4)	P1—C31—C36—C35	−177.1 (3)
P1—C11—C12—C13	178.3 (2)	C25—C26—O1—C27	−11.5 (5)
C11—C12—C13—C14	−0.9 (4)	C21—C26—O1—C27	169.7 (3)
C12—C13—C14—C15	0.2 (5)	C35—C36—O2—C37	−12.9 (5)
C13—C14—C15—C16	1.2 (5)	C31—C36—O2—C37	167.9 (3)
C14—C15—C16—C11	−1.9 (5)	C22—C21—P1—C31	−117.1 (2)
C12—C11—C16—C15	1.1 (4)	C26—C21—P1—C31	67.9 (3)
P1—C11—C16—C15	−176.8 (2)	C22—C21—P1—C11	132.1 (2)
C26—C21—C22—C23	−1.0 (5)	C26—C21—P1—C11	−42.9 (3)
P1—C21—C22—C23	−176.2 (3)	C22—C21—P1—Pd1	4.1 (3)
C21—C22—C23—C24	1.2 (5)	C26—C21—P1—Pd1	−170.9 (2)
C22—C23—C24—C25	−0.5 (6)	C32—C31—P1—C21	−2.3 (3)
C23—C24—C25—C26	−0.4 (6)	C36—C31—P1—C21	175.8 (2)
C24—C25—C26—O1	−178.1 (3)	C32—C31—P1—C11	107.8 (3)
C24—C25—C26—C21	0.6 (5)	C36—C31—P1—C11	−74.1 (2)
C22—C21—C26—O1	178.9 (3)	C32—C31—P1—Pd1	−122.1 (2)
P1—C21—C26—O1	−6.1 (4)	C36—C31—P1—Pd1	56.0 (2)
C22—C21—C26—C25	0.0 (5)	C16—C11—P1—C21	119.4 (2)
P1—C21—C26—C25	175.0 (3)	C12—C11—P1—C21	−58.5 (2)
C36—C31—C32—C33	−0.5 (5)	C16—C11—P1—C31	5.7 (3)
P1—C31—C32—C33	177.5 (2)	C12—C11—P1—C31	−172.3 (2)
C31—C32—C33—C34	−0.7 (5)	C16—C11—P1—Pd1	−119.4 (2)
C32—C33—C34—C35	1.4 (6)	C12—C11—P1—Pd1	62.7 (2)
C33—C34—C35—C36	−0.9 (6)	C21—P1—Pd1—Cl1	103.82 (10)
C34—C35—C36—O2	−179.6 (3)	C31—P1—Pd1—Cl1	−136.53 (11)
C34—C35—C36—C31	−0.3 (6)	C11—P1—Pd1—Cl1	−14.71 (10)
C32—C31—C36—O2	−179.7 (3)	C21—P1—Pd1—Cl1^i^	−76.18 (10)
P1—C31—C36—O2	2.2 (4)	C31—P1—Pd1—Cl1^i^	43.47 (11)
C32—C31—C36—C35	1.0 (5)	C11—P1—Pd1—Cl1^i^	165.29 (10)

Symmetry codes: (i) −*x*, −*y*, −*z*.
